# Using Bulky Dodecaborane-Based Dopants to Produce Mobile Charge Carriers in Amorphous Semiconducting Polymers

**DOI:** 10.1021/acs.chemmater.4c00502

**Published:** 2024-05-17

**Authors:** Yutong Wu, Charlene Z. Salamat, Alex León Ruiz, Alexander F. Simafranca, Nesibe Akmanşen-Kalayci, Eric C. Wu, Evan Doud, Zerina Mehmedović, Jeffrey R. Lindemuth, Minh D. Phan, Alexander M. Spokoyny, Benjamin J. Schwartz, Sarah H. Tolbert

**Affiliations:** † Department of Chemistry and Biochemistry, University of California Los Angeles, Los Angeles, California 90095-1569, United States; ‡ Lake Shore Cryotronics, Westerville, Ohio 43082, United States; § Center for Neutron Science, Department of Chemical and Biochemical Engineering, University of Delaware, Newark, Delaware 19716, United States; ∥ Department of Materials Science and Engineering, University of California Los Angeles, Los Angeles, California 90095-1595, United States

## Abstract

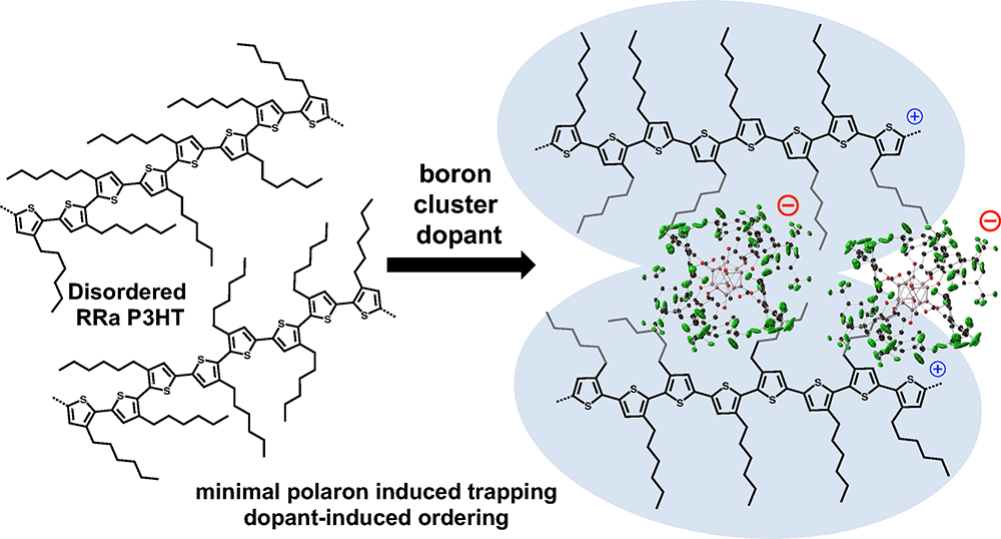

Conjugated polymers are a versatile class of electronic materials featured in a variety of next-generation electronic devices. The utility of such polymers is contingent in large part on their electrical conductivity, which depends both on the density of charge carriers (polarons) and on the carrier mobility. Carrier mobility, in turn, is largely controlled by the separation between the polarons and dopant counterions, as counterions can produce Coulombic traps. In previous work, we showed that large dopants based on dodecaborane (DDB) clusters were able to reduce Coulombic binding and thus increase carrier mobility in regioregular (RR) poly(3-hexylthiophene-2,5-diyl) (P3HT). Here, we use a DDB-based dopant to study the effects of polaron–counterion separation in chemically doped regiorandom (RRa) P3HT, which is highly amorphous. X-ray scattering shows that the DDB dopants, despite their large size, can partially order the RRa P3HT during doping and produce a doped polymer crystal structure similar to that of DDB-doped RR P3HT; Alternating Field (AC) Hall measurements also confirm a similar hole mobility. We also show that use of the large DDB dopants successfully reduces Coulombic binding of polarons and counterions in amorphous polymer regions, resulting in a 77% doping efficiency in RRa P3HT films. The DDB dopants are able to produce RRa P3HT films with a 4.92 S/cm conductivity, a value that is ∼200× higher than that achieved with 3,5,6-tetrafluoro-7,7,8,8-tetracyanoquinodimethane (F_4_TCNQ), the traditional dopant molecule. These results show that tailoring dopants to produce mobile carriers in both the amorphous and semicrystalline regions of conjugated polymers is an effective strategy for increasing achievable polymer conductivities, particularly in low-cost polymers with random regiochemistry. The results also emphasize the importance of dopant size and shape for producing Coulombically unbound, mobile polarons capable of electrical conduction in less-ordered materials.

## 1 Introduction

Semiconducting conjugated polymers have many emerging applications, including thin-film solar cells, flexible thermoelectrics, and wearable electronics.^1−3^ A wide range of semiconducting polymers have been designed for specific applications that feature various backbone structures that alter the bandgap energies;^4−8^ these design changes are also frequently accompanied by tunable side chains to aid in polymer solubility.^9−11^ As synthesized, semiconducting polymers lack intrinsic charge carriers, so doping is necessary to add the charge carriers that permit electronic conduction.^12^ Semiconducting polymers can undergo both n- and p-type doping, with the latter being more common.^13,14^ In p-type doping, electrons are transferred from the polymer’s valence band (highest occupied molecular orbital (HOMO) level) to a dopant’s lowest unoccupied molecular orbital (LUMO) level, forming a positively charged hole on the polymer backbone and a negatively charged counterion from the dopant.^15^ The hole charge carriers, along with their associated backbone deformation, are referred to as polarons. The density of charge carriers generated is related to both the oxidizing potential and the concentration of the dopant, while the carrier mobility depends on the nanoscale structure of the polymer and on Coulombic interactions with the counterions.^16−20^

The electrical conductivity of a doped polymer is determined by both the density and mobility of charge carriers, so it is important to understand how doping influences these two quantities.^8,21,22^ Strong Coulombic binding between polarons and counterions can result in localized or trapped carriers that do not significantly contribute to the conductivity; in other words, there can be instances where a dopant oxidizes the polymer chain, but the resulting carriers do not contribute to the electrical conductivity because of their very low mobility.^23−25^ In crystalline polymer regions, dopant molecules usually reside among the alkyl side chains, away from the polymer backbone.^19,26−28^ This positioning is generally desirable, as it reduces the Coulombic binding between polarons and their counterions.^17^ In disordered polymer regions, large void spaces between chains allow counterions to remain near polarons, which is partly responsible for the low conductivities observed in doped amorphous semiconducting polymers.^29^ The ability of polymer crystallites to force spatial segregation between polarons and counterions is therefore important for sample conductivity.

Many studies on regioregular (RR) and regiorandom (RRa) samples of the workhorse semiconducting polymer poly(3-hexylthiophene-2,5-diyl) (P3HT) have demonstrated that RRa P3HT is largely unable to form crystalline packing regions.^30−35^ Relative to RR P3HT, RRa P3HT has an increased bandgap energy due to breaks in conjugation at sites of polymer backbone rotation. This lowers the energy of the valence band, so that RRa P3HT is harder to dope.^36^ Poor crystallinity also causes RRa P3HT to have a low density of charge carrier percolation pathways and high Coulombic binding interactions with dopant counterions.^37,38^ All of these factors explain why doped RRa P3HT generally shows much lower conductivities than its doped RR P3HT counterpart.

In addition to polymer crystallinity, the choice of dopant is also critical to improving the electrical conductivity of doped conjugated polymers. Many dopants are small molecules that, in amorphous polymer regions, can closely associate with the polymer backbone. For example, the commonly used dopant 2,3,5,6-tetrafluoro-7,7,8,8-tetracyanoquinodimethane (F_4_TCNQ) has a flat molecular geometry that allows for π-stacking with the polymer backbone in amorphous polymer regions, producing Coulombically bound charge-transfer complexes that do not contribute to electrical conductivity.^39−41^ Even when not complexed, dopants like F_4_TCNQ still provide enough Coulombic attraction to localize nearby polarons and thus reduce carrier mobility.^42^

**Figure 1:**
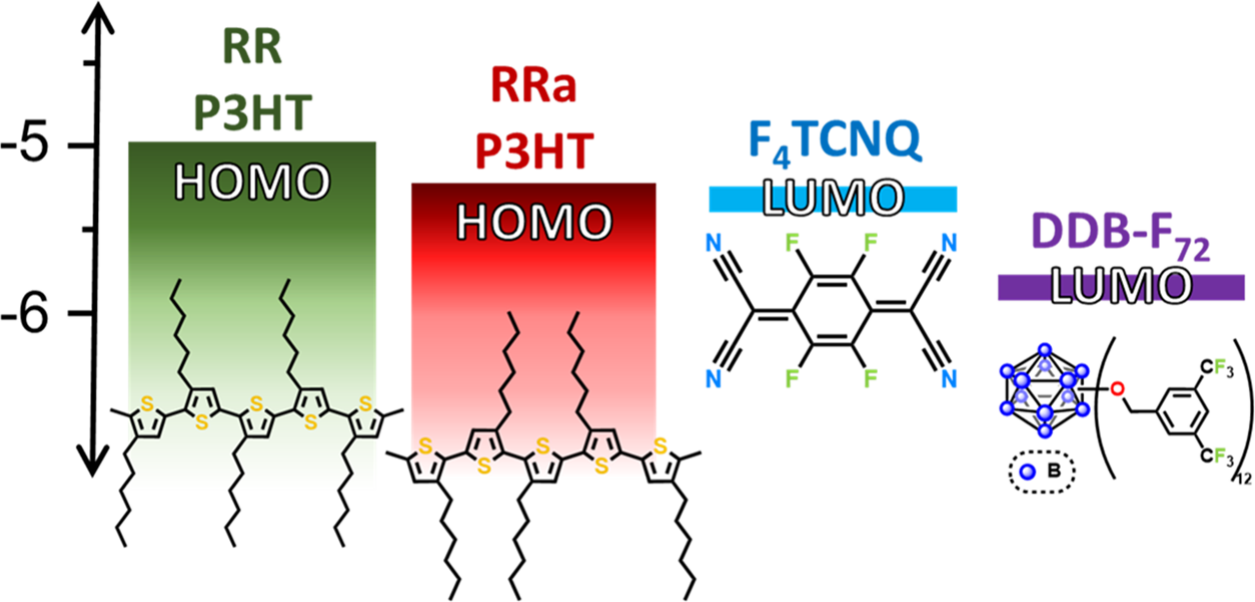
Energy level diagram of RR P3HT, RRa P3HT, and the F_4_TCNQ and DDB-F_72_ dopants. The bandgap of RRa P3HT is wider, and the valence band level is deeper than that of RR P3HT due to the amorphous nature of the polymer. RR P3HT, F_4_TCNQ, and DDB-F_72_ energy levels were taken from ref (16), and the energy level difference between RR and RRa P3HT from refs (45) and^46^

Dodecaborane (DDB) cluster-based dopants, by contrast, are a family of oxidizing agents that have been shown to successfully shield the Coulombic interaction between polaron–counterion pairs in RR P3HT due to their large size (∼2 nm in diameter).^16,17^ These dopants are composed of a icosohedral dodecaborane core on which each vertex is functionalized with a range of substituents that can be used to tune the redox potential of the molecule.^43^ A DDB cluster with 3,5-bis(trifluoromethyl)benzyloxy substituents, referred to as DDB-F_72_ (see Figure 1 for chemical structure, which contains 72 F atoms per cluster), was previously found to be an outstanding dopant for π-conjugated semiconducting systems, including carbon nanotube networks and RR P3HT.^16,17,44^ DDB-F_72_ has a reduction potential that is nearly 0.5 V lower than F_4_TCNQ, and it has been shown to achieve nearly 100% doping efficiency (i.e., one mobile carrier is produced for every dopant molecule) in RR P3HT with film conductivities routinely exceeding 10 S/cm.^16,17^

In this work, we take advantage of the fact that DDB-F_72_ can inhibit the formation of Coulombically bound polaron–counterion pairs to improve the properties of doped RRa P3HT. Using spectroscopic, electronic, and structural characterization methods, we probe structure–conductivity relationships in RRa P3HT to better understand how to improve electrical conductivity in disordered polymers, where we achieve conductivities of nearly 5 S/cm. Our results indicate that large dopants like DDB-F_72_ can facilitate electronic transport in noncrystalline polymers in three ways: their high oxidation potential allows for high carrier densities, their large size prevents Coulombic trapping despite polymer disorder, and they are able to improve polymer ordering through doping-induced crystallization.

## 2 Results and Discussion

**Figure 2:**
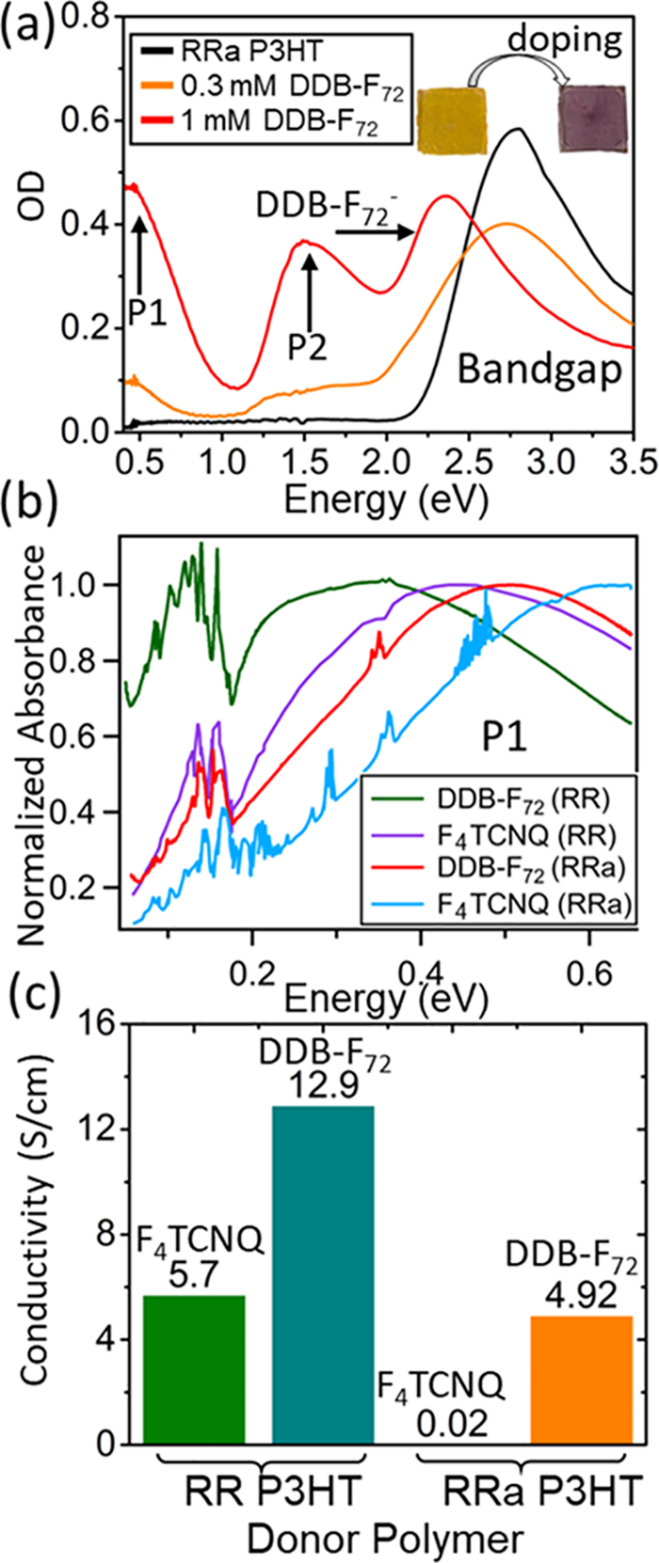
(a) UV–vis-IR absorbance spectra of RRa P3HT doped with low and high concentrations of DDB-F_72_. Doping is characterized by the bleaching of the P3HT neutral peak (∼2.8 eV) corresponding to the bandgap transition and the appearance of polaron transitions in the red and IR ranges (designated P1 and P2) and the DDB-F_72_ anion absorption in the visible. (b) Normalized FT-IR spectra of the P1 transition of RR and RRa P3HT doped with DDB-F_72_ and F_4_TCNQ. The position of the P1 transition has been shown to reflect the degree of Coulomb binding of the polaron to the counterion, where a lower energy P1 transition indicates reduced Coulombic binding. (c) Measured conductivities of RR and RRa P3HT doped with DDB-F_72_ and F_4_TCNQ at a dopant concentration of 1 mM. For both polymers, DDB-F_72_ produced higher conductivities than F_4_TCNQ.

To understand the doping of RR and RRa P3HT with DDB-F_72_, we must first consider the relative polymer band and dopant orbital energies involved. Figure 1 shows the HOMO levels for RR P3HT and RRa P3HT as well as the LUMO levels of F_4_TCNQ and DDB-F_72_. The RRa bandgap energy of ∼2.8 eV appears as a strong absorbance peak in undoped RRa P3HT, as seen by the black curve in Figure 2a. As RRa P3HT is doped with DDB-F_72_, the removal of electrons from the valence band and the creation of intragap states causes the intensity of the bandgap transition to decrease and new peaks corresponding to polaronic transitions (P1 and P2) to appear.^47^ Visually, these changes in absorbance upon doping result in a color change of the RRa P3HT film from yellow/orange when undoped to purple when doped (Figure 2a, inset). For the 1 mM DDB-F_72_ doping solution (red curve), the bandgap transition peak is largely absent, and an absorption peak at 2.4 eV corresponding to the DDB-F_72_ anion becomes readily evident, indicating that the film is highly doped.^16,17^ See Figure S1 for data on more DDB-F_72_ concentrations.

In our previous work, we studied the doping of RR P3HT with DDB-F_72_ and other dopants.^16,17^ For RR P3HT, we demonstrated that the more a polaron is Coulombically bound to its counterion, the more blue-shifted its P1 absorption peak is.^16,20,39^ The P1 absorption peaks for DDB-F_72_-doped RR and RRa P3HT are shown in Figure 2b. The P1 peaks for F_4_TCNQ-doped RR and RRa P3HT from our previous work are also included for comparison.^40^ The higher P1 transition energies for doped RRa P3HT versus RR P3HT arise from the larger bandgap energy of RRa P3HT. RRa P3HT is an amorphous polymer, so independently of the Coulomb binding, the polarons in this material are inherently less delocalized than those in RR P3HT, explaining why the polarons in RRa P3HT have a blue-shifted P1 absorption. For both polymers, the P1 transition energies are significantly lower for the DDB-F_72_-doped films relative to the F_4_TCNQ-doped films of the same regioregularity, indicating reduced Coulombic binding between the polaronic holes on P3HT and DDB-F_72_ anions in both polymer types. This is a direct result of the size of the DDB-F_72_ dopant; with its ∼2 nm diameter, DDB-F_72_ effectively separates polarons on the polymer backbone from the counterion charge.^17^ The DDB-F_72_ dopant is therefore a good choice for doping RRa P3HT, where the lack of crystallinity decreases polaron delocalization and increases the Coulombic binding of charge carriers.

Although the DDB-F_72_ dopant has been shown to produce high conductivities in RR P3HT,^17^ conductivities from DDB-F_72_-doped RRa P3HT have not been previously reported. Figure 2c shows the conductivities of both RR and RRa P3HT films doped by sequential processing (SqP)^16,19,20^ with both F_4_TCNQ and DDB-F_72_. The conductivities of both RR and RRa P3HT films doped with DDB-F_72_ are higher than those doped with F_4_TCNQ, in line with the reduced Coulombic binding spectroscopically observed with DDB-F_72_. Surprisingly, DDB-F_72_ improves the conductivity of RR P3HT by only a factor of ∼2, while RRa P3HT conductivities with DDB-F_72_ are ∼200× higher than those doped with F_4_TCNQ. To explain this, we show that this dramatic improvement in sample conductivity reflects both the ability of DDB-F_72_ to dope the amorphous regions of RRa P3HT, and therefore increase the fraction of the film used in conduction, and the ability of DDB-F_72_ to partially order the RRa P3HT by using its large size to fill what would be large gaps in a crystalline RRa film. See Supporting Information (SI) Section S1 for details of conductivity measurements and data at lower doping levels.

To identify whether the observed improvements in conductivity were due to increased carrier density or carrier mobility, we used AC Hall effect measurements to determine the density of mobile carriers (*n*_H_) and their mobilities (μ_H_). These values are summarized in Table 1.^20,39,48,49^ Despite the ability of AC-field measurements to successfully produce a Hall voltage in low-mobility organic materials, there are still challenges in the interpretation of such measurements. This is because in organic semiconductors, carriers that move by hopping can respond to the transverse Hall electric field and thus drift in the opposite direction relative to carriers with band-like transport that experience the Lorentz force from the external magnetic field. Thus, instead of the standard band-transport Hall voltage, *V*_Hall_, given by *en = IB*/*V*_Hall_ (here, *e* is the electric charge, *n* is the carrier density, *I* is the current, and *B* is the applied magnetic field), when carriers move by hopping, the standard expression produces an incorrectly large carrier concentration and therefore an incorrectly low mobility. Podzorov and co-workers,^50^ as well as Sirringhaus and co-workers,^51^ have worked on rationalizing the observed Hall voltages when there is a mixture of hopping and band-like transport, although unfortunately, there are too many unknown parameters for us to rigorously extract the fraction of carriers that move by band-like and hopping transport in the films studied here. Nonetheless, the trend between higher mobility and a more structured polymer film is followed throughout.

Slightly lower doping levels were used for the AC Hall measurements compared to those presented in Figure 2, both because higher doping levels decrease the measured Hall voltage and increase the error and because lower doping levels produce flatter films, which is important for the matched neutron reflectometry data discussed below. We also used different DDB-F_72_ concentrations for the RRa (at 0.85 mM DDB-F_72_) and RR (at 0.3 mM DDB-F_72_) samples in an attempt to achieve similar carrier densities. Surprisingly, the hole mobilities of both RR and RRa P3HT doped with DDB-F_72_ at these doping conditions are nearly identical. This equivalence is quite unexpected for a disordered polymer like RRa P3HT. The higher conductivity of the DDB-F_72_-doped RR P3HT compared to the RRa system in Figure 2 must then result mostly from a higher carrier density for the RR P3HT compared to the doped RRa P3HT. This result is reasonable given that RRa P3HT is several hundred mV harder to oxidize than RR P3HT (Figure 1). Although the DDB-F_72_ dopant is able to produce high carrier mobilities from RRa P3HT, it is ultimately limited in carrier production by its oxidizing potential relative to the low-lying valence band of RRa P3HT.

**Table 1:** Electronic Properties of DDB-F_72_-Doped RR and RRa P3HT^a^

polymer	[DDB-F_72_] (mM)	polymer SLD^b^ (Å^–2^)	dopant density^b^ (cm^–3^)	μ_H_^c^ (cm^2^V^–1^S^–1^)	σ^c^ (S cm^–1^)	*n*_H_^c^ (cm^–3^)	doping efficiency^d^ (%)
RRa P3HT	0.85	0.45 × 10^–2^	2.5 × 10^20^	0.098 ± 0.01	3.0 ± 0.3	(1.91 ± 0.4) × 10^20^	77
RR P3HT^16^	0.3	0.56 × 10^–2^	5.3 × 10^20^	0.084	6.8 ± 0.5	5.08 × 10^20^	96

a Calculations are detailed in the SI.

b Calculated from fitted NR measurements.

c Calculated from AC Hall Effect measurements.

d Estimated by comparing the obtained dopant density and carrier density values.

**Figure 3:**
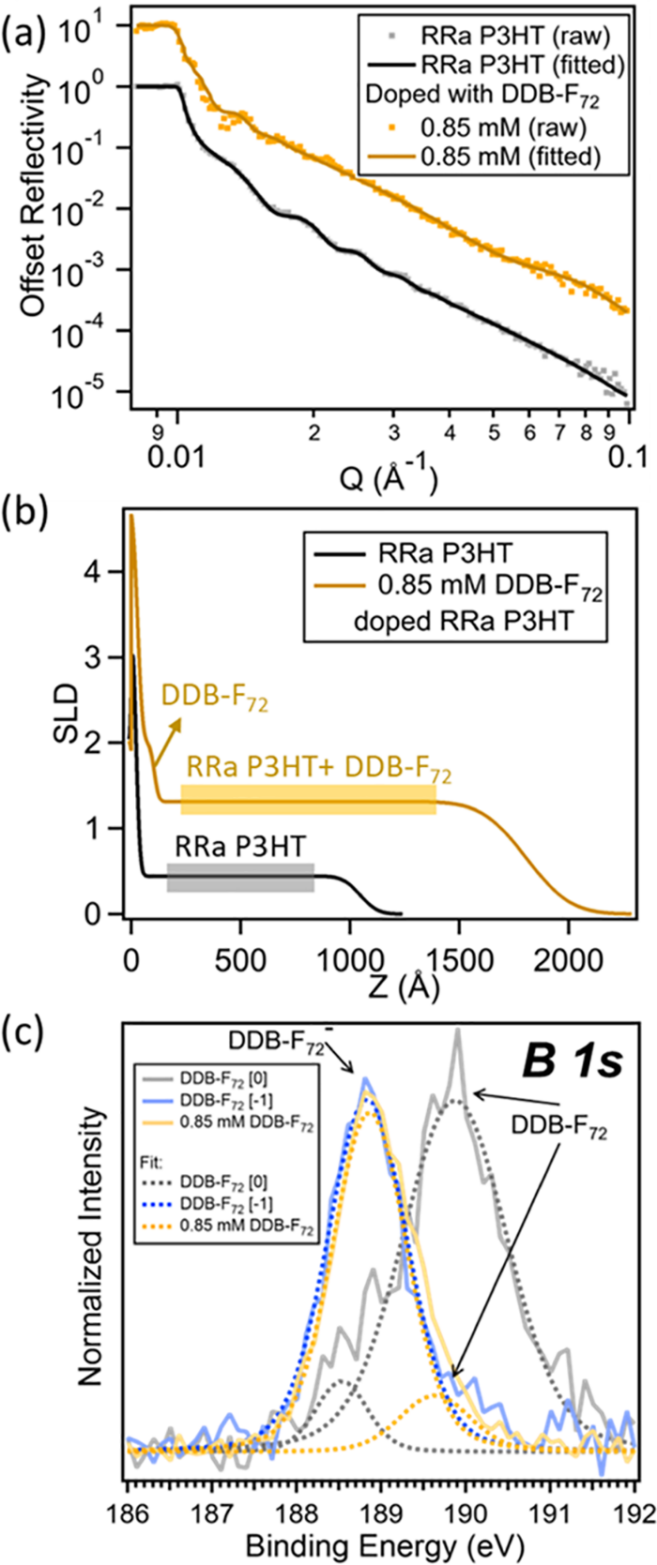
(a) Data (dots) and fits (solid curve) for neutron reflectometry (NR) profiles of undoped RRa P3HT (black trace) and RRa P3HT doped with 0.85 mM DDB-F_72_ (yellow trace). (b) SLD profiles of undoped RRa P3HT (black trace) and RRa P3HT doped with 0.85 mM DDB-F_72_ (yellow trace). Other than a slight buildup of DDB-F_72_ near the substrate surface (*Z* = 0 Å), DDB-F_72_ distributes uniformly in the bulk of the RRa P3HT film. (c) XPS data collected on neutral DDB-F_72_ (gray/black traces), DDB-F_72_ anion (blue traces), and RRa P3HT doped with 0.85 mM DDB-F_72_ (yellow traces), with the data shown as solid lines and the fits shown as dashed lines. The yellow curve indicates that the DDB-F_72_ in the RRa P3HT film is dominantly in the anionic form but that some neutral DDB-F_72_ is also present.

Although the large size of the DDB-F_72_ dopant is good for reducing Coulombic binding forces, it also impedes dopant diffusion into P3HT films, so it is necessary to verify that the dopant can penetrate and dope the entirety of the polymer film volume. Neutron reflectometry (NR) measurements were performed on DDB-F_72_-doped RR and RRa P3HT films to characterize the distribution of dopant molecules in the polymer films. The raw NR data and corresponding fit curves are shown in Figures 3a and S2a. The reflectometry data were fit based on measured scattering length densities (SLDs) for RRa P3HT (measured at 0.45 × 10^–6^ Å^–2^) and DDB-F_72_ (calculated at 0.56 × 10^–6^ Å^–2^)^16^ to determine the relative composition of the polymer and the dopant.^50,52^Figures 3b and S2b show the fitted film SLD as a function of distance from the substrate, where *Z* = 0 is the substrate–film interface and the SLD approaches 0 near the film–air interface. A slight buildup of DDB-F_72_ is observed near the substrate surface in the doped RRa P3HT film, indicating excellent DDB penetration into the film. Other than that small buildup, the SLD of the doped film is constant and uniformly higher than the undoped RRa P3HT film, indicating that the DDB-F_72_ dopant is distributed evenly throughout the rest of the film. The doped film is also shown to have increased in thickness due to volume expansion from dopant infiltration. Together, these results indicate that DDB-F_72_ is not diffusion-limited in its ability to dope.

The quantitative nature of the SLD fits allows us to calculate a doping efficiency—the ratio of the number of mobile carriers that contribute to conductivity (as determined from AC Hall measurements) to the number of dopant molecules present in the film. Dopant number densities were extracted from the NR SLDs, and mobile carrier density numbers for the films were calculated from AC Hall effect measurements using the same method as in our previous study,^16^ as described in the SI. These values were combined to calculate doping efficiencies for DDB-F_72_-doped RR and RRa P3HT. Table 1 shows the calculated doping efficiencies, which are 96^16^ and 77% for RR and RRa P3HT, respectively.

The lower doping efficiency for RRa P3HT may be partially due to its lower valence band energy (Figure 1), reducing the oxidizing potential of the RRa P3HT and DDB-F_72_ pair and allowing some unreduced DDB-F_72_ to remain in the film. To test the validity of this hypothesis, X-ray photoelectron spectroscopy (XPS) data was collected to investigate the oxidation state of boron near the top surface of the film. Figure 3c shows the B 1s spectra of the neutral DDB-F_72_ cluster^17^ (black/gray traces), the DDB-F_72_ anion^17^ (blue traces), and RRa P3HT doped with 0.85 mM DDB-F_72_ (yellow traces). The data indicate that DDB-F_72_ clusters in the polymer film are mostly in the anionic form but that some neutral DDB-F_72_ is also present in the doped RRa P3HT. The fits indicate that the B 1s peak is composed of ∼86% anionic species and 14% neutral species, while in the RR P3HT doping case, the B 1s peak is composed entirely of the anionic species.^17^ This, in combination with the presence of some trapped carriers in the doped RRa P3HT, which are trapped not by Coulomb attraction to their counterions but by polymer chain disorder, results in the lower doping efficiency for RRa P3HT doped with DDB-F_72_. Despite small differences, the doping efficiency values for both RR and RRa P3HT are quite high, indicating that most of the DDB-F_72_ molecules detected within the polymer films produced mobile carriers, reinforcing the idea that reducing dopant-polaron Coulombic binding is key to high doping efficiency.^20^

Although low Coulombic binding between DDB-F_72_ and generated polarons provides a partial explanation for the high carrier mobility in doped RRa P3HT, it is surprising that the carrier mobility of an initially amorphous polymer like RRa P3HT is so similar to that of semicrystalline RR P3HT when doped. Because carrier mobility is closely linked with film structure, an explanation may lie in structural similarities between the doped RR and RRa P3HT films. To investigate this, a combination of grazing incidence small- and wide-angle X-ray scattering (GISAXS and GIWAXS, respectively) techniques were employed to probe the structural changes that occur during doping with DDB-F_72_.

**Figure 4:**
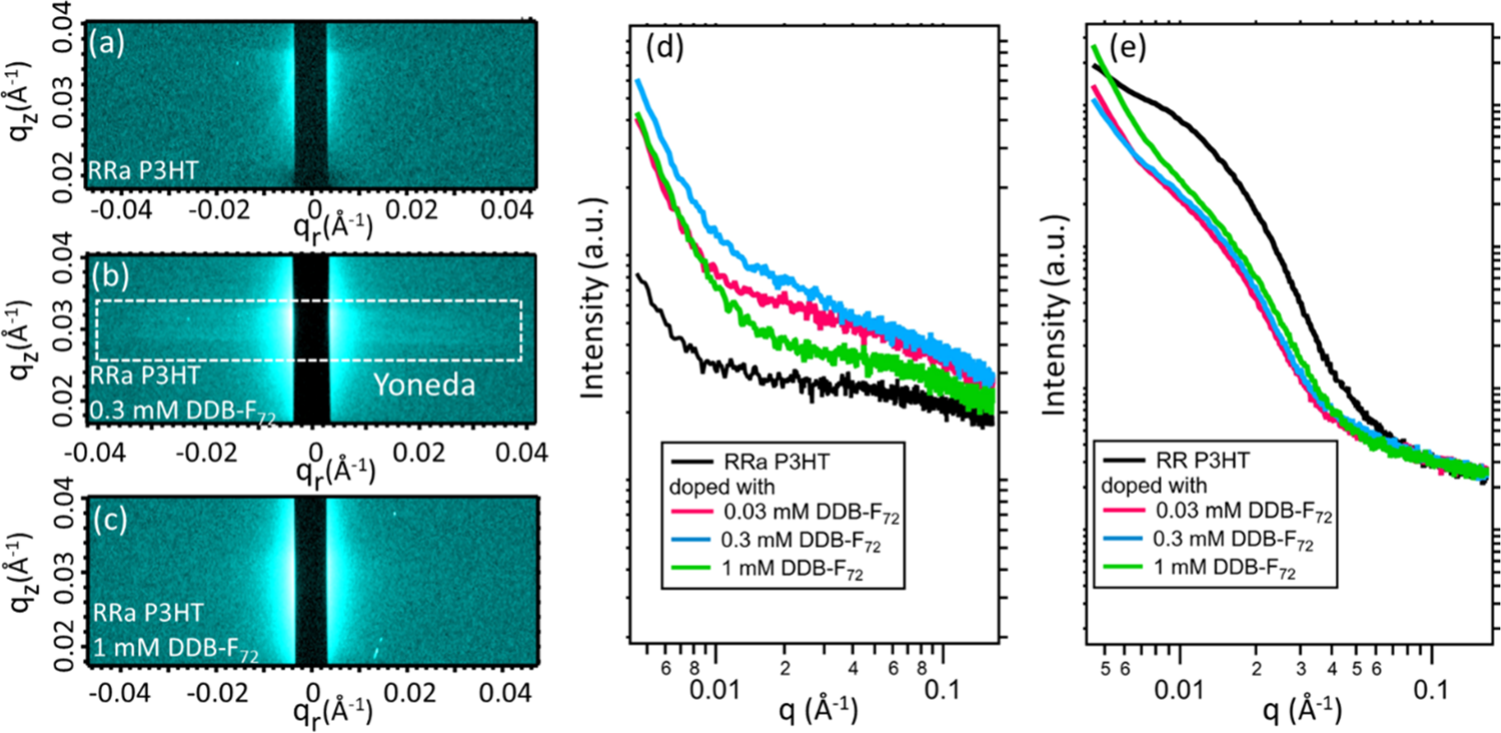
2D GISAXS patterns of (a) RRa P3HT, (b) RRa P3HT doped with 0.3 mM DDB-F_72_, and (c) RRa P3HT doped with 1 mM DDB-F_72_. Doping with low concentrations of DDB-F_72_ produced the horizontal Yoneda peak (boxed), indicating the introduction of electron density contrast at small length scales. Further doping is shown to erase the Yoneda peak, indicating the disappearance of electron density contrast. Integrated Yoneda regions for (d) RRa and (e) RR P3HT doped with DDB-F_72_ at three varying concentrations. Doping with DDB-F_72_ results in a decrease in the intensity of the Yoneda peak and a shift to higher *q* values, corresponding to disruption of polymer crystallites upon doping. In RRa P3HT (d), DDB-F_72_ doping increases the scattering significantly at low (pink trace) and medium (blue trace) dopant concentrations, producing a well-defined Yoneda peak. At high dopant concentrations, scattering intensity is reduced, indicating a loss of domain contrast.

First, changes in the crystallite-scale structure of DDB-F_72_-doped RR and RRa P3HT films were observed through GISAXS studies. Figures 4a–c, S3 and S4 show two-dimensional (2D) GISAXS patterns, zoomed in to emphasize the Yoneda peak region. The Yoneda peak is a horizontal scattering peak produced by enhanced X-ray scattering near the angle of internal reflection at the polymer–air interface that contains information on the lateral electron density contrast in the thin film.^53,54^ Horizontal integration of the Yoneda peak region of undoped and DDB-F_72_-doped RRa P3HT (Figure 4d) shows a large increase in scattering at lower *q* values (larger correlation distances, *d*), corresponding to the creation of small polymer domains that are likely crystalline in nature. Scattering at higher *q* values also increases slightly, suggesting a range of crystallite sizes. At high doping levels (green trace, 1 mM DDB-F_72_), scattering at low *q* values decreases slightly, likely due to a loss of domain contrast as the film further crystallizes.

The integrated Yoneda region for undoped RR P3HT (Figure 4e) contains a peak at ∼0.01 Å^–1^ (*d* ∼ 60 nm). Upon dopant intercalation, this peak shifts to a larger *q* value (*q* ∼ 0.015 Å^–1^; *d* ∼ 42 nm) and decreases in scattering intensity. Previous work with the DDB-F_72_ dopant shows that the large dopant molecule intercalates into the lamellar stacks of P3HT.^16,17^ The shift of the observed peak to larger *q* (smaller *d*) is believed to indicate the breaking up of existing crystallites into smaller regions of varying sizes due to the strain of intercalation by a large dopant. At high doping levels (green trace, 1 mM DDB-F_72_), an increase in low *q* scattering is observed. Based on the appearance of crystalline regions in RRa P3HT during doping, the DDB-F_72_ dopant must be capable of converting amorphous P3HT to crystallites. This increase in low *q* scattering is therefore believed to correlate with the expansion of crystallites into the surrounding amorphous regions, producing larger crystallites than those present at low dopant concentrations.

**Figure 5:**
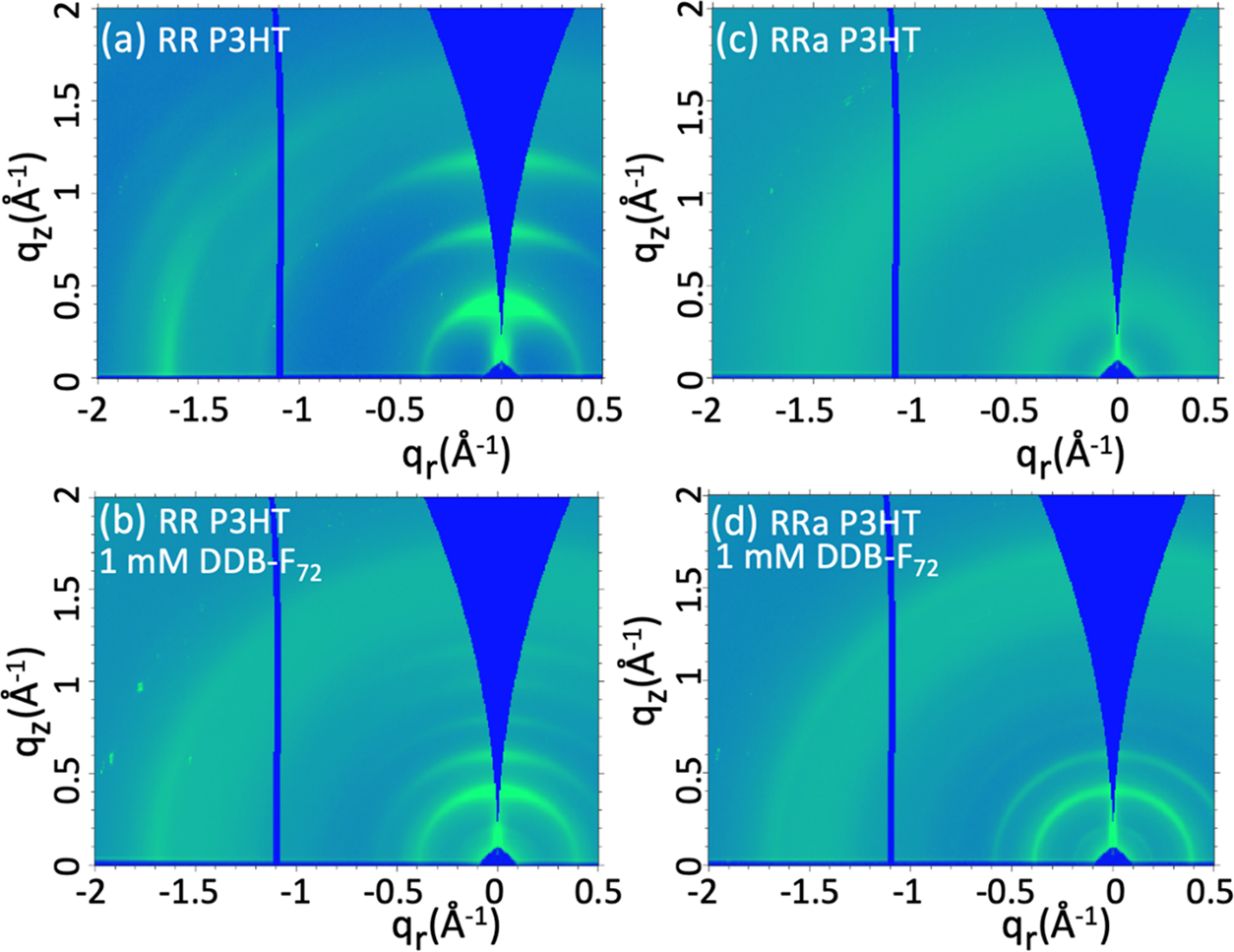
2D GIWAXS patterns of (a) RR P3HT, (b) RR P3HT doped with 1 mM DDB-F_72_, (c) RRa P3HT, and (d) RRa P3HT doped with 1 mM DDB-F_72_. Doping with DDB-F_72_ induces crystallinity in RRa P3HT polymer films, producing similar crystalline structures in both doped RR and RRa P3HT.

**Figure 6:**
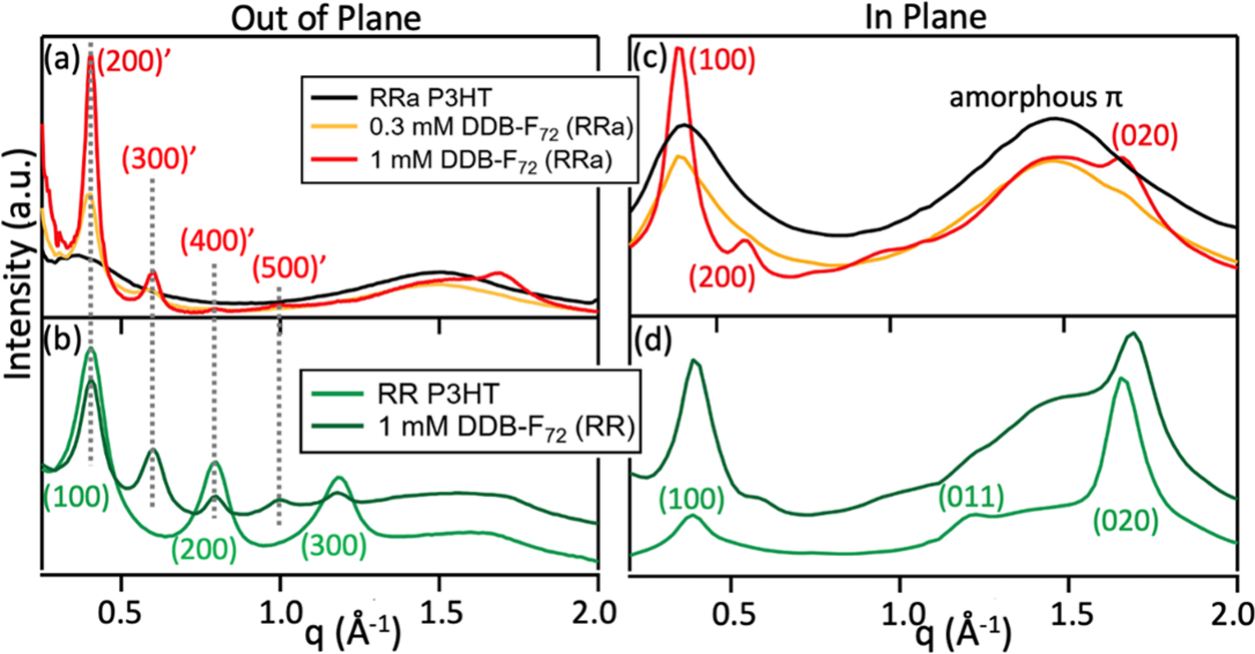
Radially integrated out-of-plane and in-plane GIWAXS patterns for (a, c) RRa P3HT and (b, d) RR P3HT doped with DDB-F_72_, respectively. Doped RR and RRa P3HT show identical lamellar diffraction peak positions, indicating identical crystalline structures within the doping-induced crystalline regions of RRa and RR P3HT.

Next, GIWAXS studies were performed to observe intracrystallite differences in structure.^55^Figures 5a–d and S5 show 2D GIWAXS patterns for undoped and DDB-F_72_-doped RR and RRa P3HT; the corresponding one-dimensional (1D) patterns produced by radial integration near the *q*_*r*_- and *q*_*z*_-axes (“in-plane” and “out-of-plane” directions, respectively) are plotted in Figure 6. As is well-known, undoped RR P3HT (Figure 5a) has an “edge-on” orientation, with the alkyl side chains of the polymers oriented near normal to the substrate surface and the polymer backbone and π-stacks lying in the plane of the substrate.^56−58^ The layers of polymer backbone separated by the side chains produce out-of-plane scattering peaks at multiples of *q*_*z*_ ∼ 0.4 Å^–1^, corresponding to a *d*-spacing of ∼16 Å, which we refer to as the lamellar spacing, *d*_lam_. The π-stacking of the thiophene rings produces scattering peaks in the plane of the substrate at *q*_*r*_ ∼ 1.65 Å^–1^, corresponding to a *d*-spacing of ∼3.81 Å, which we refer to as *d*_π_.^9^ The broad peak centered at *q*_*r*_ ∼ 1.5 Å^–1^ was confirmed by molecular modeling to correspond to disordered π-stacking distances caused by random thiophene ring stacking in the disordered regions of the polymer film (Figure S6).

In contrast, undoped RRa P3HT (Figure 5c) shows only two diffuse and isotropic scattering rings, one corresponding to the lamellar spacing (i.e., alkyl side chain spacing, *q* centered at ∼0.4 Å^–1^; *d*_lam_ ∼ 16 Å) and the other corresponding to the amorphous π-stacking discussed above (*q* centered at ∼1.5 Å^–1^; *d*_π_ ∼ 4.19 Å). The isotropic nature of the rings indicates a lack of specific crystallite orientation relative to the substrate. The broadness of the isotropic rings and the larger π-stacking distance of RRa P3HT both suggest a high level of disorder in the polymer chain packing, as expected for an initially highly amorphous material.^29^

Upon doping RR P3HT with DDB-F_72_ (Figures 5b and 6b,d), the lamellar distance doubles (scattering peaks at multiples of *q*_*z*_ ∼ 0.2 Å^–1^; *d*_lam_ ∼ 31 Å) to accommodate the intercalation of the very large DDB cluster into the alkyl side chain region,^16^ while the π-stacking distance (*q*_*r*_ ∼ 1.70 Å^–1^; *d*_π_ ∼ 3.70 Å) becomes slightly contracted. Surprisingly, doping of RRa P3HT with DDB-F_72_ produces a set of dopant-intercalated lamellar and π-stacking peaks (Figures 5d and 6) that are identical to those of doped RR P3HT. This indicates that the amorphous RRa P3HT undergoes dopant-induced ordering, resulting in ordered crystallites, with a doped crystal structure that is the same as that of doped RR P3HT.^31,34,59^

The presence of higher-order lamellar overtones and sharper lamellar peaks in the integrated patterns for doped RRa P3HT compared to RR P3HT (Figure 6) indicate that RRa P3HT has larger doped crystallites than RR P3HT. However, the overtones fade much faster in RRa P3HT than RR P3HT (Figure 6a,b), indicating that while RR P3HT crystallites may be smaller, they retain a higher level of paracrystalline order than the RRa P3HT polymer,^60,61^ as determined by GISAXS. The more isotropic (i.e., ring-like) lamellar peaks observed in doped RRa P3HT (Figure 5d) are consistent with crystallites that nucleated in the bulk of the polymer film away from the lattice-templating effects of the substrate. It is important to note that a large amorphous π-stacking peak remains, even in the most highly doped RRa samples, indicating that although some highly crystalline domains are created, large fractions of the RRa P3HT film remains amorphous, making the high mobility values observed here all the more unique.

Doping-induced crystallization has been observed previously in RRa P3HT doped with F_4_TCNQ,^31,34^ so the fact that it also occurs upon doping with DDB-F_72_ is not unexpected. What is unexpected is that the final structure shows such narrow diffraction peaks, albeit with high paracrystalline disorder. The crystalline structure observed upon doping RRa P3HT with F_4_TCNQ is less ordered.^31,34^ This suggests that the DDB-F_72_ anion may be ideally sized to fit within the cavities created by the random regiochemistry. The large size of the DDB-F_72_ cluster is usually considered disadvantageous for producing highly crystalline polymers,^16^ but for amorphous materials like RRa P3HT, it may actually be an advantage.

## 3 Conclusions

Here, we have demonstrated an effective strategy for the doping of RRa P3HT. The dodecaborane cluster-based large molecular dopant DDB-F_72_ was shown to dope RRa P3HT with a 77% doping efficiency and to induce partial crystallization of RRa P3HT in the process. The doped RRa P3HT film had a conductivity value of 4.9 S/cm, 200× higher than the conductivity of F_4_TCNQ-doped RRa P3HT and only 2.5× lower than that of DDB-F_72_-doped RR P3HT. Despite the fact that RRa P3HT films start in a highly amorphous state, structural comparisons of DDB-F_72_-doped RR and RRa P3HT films revealed close similarities in the crystalline structure of both doped films, although the doped RRa films retain a large fraction of disordered polymer. Remarkably, similar hole mobilities are measured for both polymer regioregularities, showing the power of using dopant counterions that are large enough to prevent Coulomb trapping of polarons, even in disordered film regions. Through careful selection of a dopant that both reduces Coulombic binding of polarons and induces crystallite formation, we have shown that initially amorphous semiconducting polymers can be made to have similar conductivities to standard semicrystalline polymers, potentially opening the door to higher conductivity applications for low-cost, disordered conjugated polymers.

## 4 Experimental Methods

### 4.1 Materials

Regiorandom (RRa) poly(3-hexylthiophene-2,5-diyl) (P3HT) (Rieke Metals Inc., *M*_w_ = 30–90 K) and regioregular (RR) P3HT (4002-EE, Rieke Metals Inc., *M_n_* = 50–70 K, regioregularity 91–94%, polydispersity 2.0–2.5) were used as purchased. The dopant molecule DDB-F_72_ was synthesized in-house following an established procedure.^62^

### 4.2 Film Fabrication

Glass and silicon substrates were cleaned by sonicating in Alconox detergent aqueous solution, acetone, and isopropanol sequentially for 15 min each, followed by plasma cleaning using a Harrick plasma cleaner PDC-32G for 15 min. Film fabrication was carried out in a glovebox under nitrogen. RR P3HT and RRa P3HT films were spin-coated at 1000 rpm for 60 s from 20 mg/mL polymer solutions in 1,2-dichlorobenzene (ODCB, Sigma-Aldrich, anhydrous, 99%). Film thickness measurements were taken on a Dektak profilometer. The DDB-F_72_ dopant was applied to the precast P3HT films by solution sequential processing (SqP) from *n*-butyl acetate (*n*-BA, Fisher Scientific, reagent grade, dried by stirring with magnesium sulfate and subsequent distillation) at the stated concentrations. Once applied, the dopant solution soaked the polymer films for 20 s before spin-coating at 4000 rpm for 10 s. Previous work has demonstrated that the SqP method effectively delivers the DDB-based dopants throughout the polymer films.^16,17^ Various in-house experiments have been conducted, where the DDB-F_72_ doping solution soaked RRa P3HT films at different times. At 20 s soaking time, there are no more significant changes to the doping, and thus, we can say that at *t* > 20 s, the kinetics are mostly invariant.

### 4.3 Optical Spectroscopy

Ultraviolet–visible (UV–vis)-NIR absorption spectra were acquired from 300–3000 nm using a Shimadzu UV3101PC scanning spectrophotometer for films prepared on glass substrates. Fourier transform infrared (FT-IR) data was acquired from 220–7000 cm^–1^ for matched samples prepared on KBr plates using a Jasco FT/IR-420 spectrometer.

### 4.4 Conductivity Measurements

Conductivity measurements were taken on 1.5 cm × 1.5 cm glass substrates with thermally evaporated silver contacts placed at the corners of the substrates. Sheet resistance measurements were taken using the van der Pauw technique with a Keithley 2400 SourceMeter controlled by Labview software. The max current sourced was held to 1 mW. Reported conductivity values are the average of at least three distinct samples. See Supporting Information section 1 and Table S1 for more detail and examples of individual measurements.

### 4.5 Neutron Reflectometry (NR) Measurements

Reflectivity measurements were performed on the liquids reflectometer (LIQREF), BL-4B, at the Spallation Neutron Source (SNS) of the Oak Ridge National Laboratory (ORNL) with a 2D position-sensitive ^3^He detector. A 3.4 Å bandwidth, extracted from a wavelength range of 2.55–16.70 Å, was used at measurement angles of 0.60 and 1.19° to attain a *q*-range of 0.008 to 0.102 Å^–1^. The measured films were fabricated on 1.5 cm × 1.5 cm silicon substrates (B-doped, p-type, ⟨100⟩ oriented) using 0.85 mM dopant solutions by SqP. The beam footprint was kept constant through adjustments in the slit opening commensurate with the angle of incidence. Data reduction was done using RefRed, and subsequent analysis was performed on ORNL’s web interface (Webi) using the Refl1D Python package.^63^

To fit the NR data, the free parameters of layer thickness, scattering length density (SLD), and layer roughness were given estimated ranges that were optimized by a built-in machine learning algorithm. Extra layers were added or removed based on the resulting fit parameters.^63^ To remove any potential bias in the fits, numerical fittings were performed independently by two people before comparing the results. The SLD contributions of the DDB-F_72_ dopant and the RRa P3HT matrix to the active layer SLD were taken from a calculated SLD for the DDB-F_72_ molecule and a measured SLD for pure RRa P3HT.

### 4.6 Grazing Incidence Wide-Angle X-ray Scattering (GIWAXS)

Films for GIWAXS measurements were prepared on 1 cm × 1 cm single-crystal silicon substrates (B-doped, p-type, ⟨100⟩ oriented). Measurements were performed at the Advanced Photon Source (APS) on beamline 8-ID-E with a 10.92 keV X-ray beam incident at 0.133° and a detector distance of 217 mm. Calibration and gap-filling of the obtained 2D diffractograms were performed using the MATLAB toolbox GIXSGUI. Radial integration between 0 and 10° (out-of-plane) and 80–90° (in-plane) relative to the positive sample *z*-axis as well as baseline corrections and peak fitting were also performed in GIXSGUI.^64^

### 4.7 Grazing Incidence Small-Angle X-ray Scattering (GISAXS)

Films for GISAXS measurements were prepared on 1.5 cm × 1.5 cm single-crystal silicon substrates (B-doped, p-type, ⟨100⟩ oriented). Measurements were performed at the Stanford Synchrotron Radiation Lightsource (SSRL) on beamline 1–5 using a wavelength of 1.0332 Å at an incident angle of 0.14°. 2D GISAXS patterns were calibrated and analyzed using the Nika package on Igor Pro 8.^65^ 1D GISAXS patterns were calculated by vertically integrating the Yoneda peak, or the high-intensity scattering near the critical angle of the polymer film due to the Vineyard effect.^66^

### 4.8 Alternating Field (AC) Hall Effect Measurements

Doped P3HT films for AC Hall Effect measurements were made on 1 cm × 1 cm glass substrates. Following film fabrication, silver electrodes were thermally evaporated on the corners of the samples with an Angstrom Engineering, Inc. evaporator at a pressure <1 μTorr and a deposition rate of 0.5 Å/s up to 10 nm, followed by 1 Å/s to a final thickness of 60 nm. Samples were packaged in scintillation vials under an argon atmosphere before being sent for testing. AC Hall measurements were performed with a Lake Shore model 8400 series AC Hall probe system at a field strength of 0.6484 T and a current of 10.0 μA under flowing nitrogen. The instrument allows the Hall voltage to be readily distinguished from the static misalignment offset voltage, which can be quite large in low-mobility materials.

### 4.9 Simulation for RR and RRa P3HT

MD simulations were performed using GROAMCS, and the force field used was developed by Wildman et al.^67^ The simulation box consists of 72 chains of P3HT with 24 monomers. The positions of the hexyl side chain for each monomer were chosen at random. Structure factors were calculated using the GROMACS’ build-in function. Detailed procedures can be found in the SI.

## Supporting Information Available

Experimental details; NR and doping efficiency calculations; GIWAXS of P3HT films doped with DDB-F_72_ at various dopant concentrations; crystallinity change of F_4_TCNQ- and DDB-F_72_-doped RRa P3HT; GISAXS of DDB-F_72_-doped P3HT; UV–vis and conductivity data at varying DDB-F_72_ doping concentrations; and molecular dynamics simulation for RR and RRa P3HT; along with details of the simulation methods (PDF)
