# Supplementary material for: Using Bulky Dodecaborane-Based Dopants to Produce Mobile Charge Carriers in Amorphous Semiconducting Polymers
Source: Chem Mater. 2024 May 17;36(11):5552–62. doi: 10.1021/acs.chemmater.4c00502 (PMC11171275; doi:10.1021/acs.chemmater.4c00502)
Supplement: Supplementary file 1 — cm4c00502_si_001.pdf [file cm4c00502_si_001.pdf]

Supporting Information

Using Bulky Dodecaborane-Based Dopants to Produce Mobile Charge Carriers in Amorphous Semiconducting Polymers

Yutong Wu[1,‡], Charlene Z. Salamat[1,‡], Alex León Ruiz[1], Alexander F. Simafranca[1], Nesibe Akmanşen-Kalayci[1], Eric C. Wu[1], Evan Doud[1], Zerina Mehmedović[1], Jeffrey R. Lindemuth[2], Minh D. Phan[3], Alexander M. Spokoyny[1], Benjamin J. Schwartz[1,*], Sarah H. Tolbert[1,4*]

[‡]These authors contributed equally to this work
[*]Address correspondence to tolbert@chem.ucla.edu, schwartz@chem.ucla.edu

[1] Department of Chemistry and Biochemistry, University of California Los Angeles, Los Angeles, CA 90095-1569, USA

[2] Lake Shore Cryotronics, Westerville, OH 43082, USA

[3] Center for Neutron Science, Department of Chemical and Biochemical Engineering, University of Delaware, Newark, DE, 19716, USA

[4] Department of Materials Science and Engineering, University of California Los Angeles, Los Angeles, CA 90095-1595, USA

Table of Contents

1. Additional Conductivity Measurements 2
2. Neutron Reflectometry (NR) & Doping Efficiency Calculation 3
3. GISAXS for DDB-F_{72} doped P3HT 4
4. GIWAXS for P3HT films doped with DDB-F_{72} at various dopant concentrations 5
5. Simulation Details for RR and RRa P3HT 6
   5.1 Additional Simulation Methods 6

1. Additional Conductivity Measurements

Table S1. Van der Pauw conductivity measurements of 1 mM DDB-F_{72} doped RRa P3HT films

Sample #	R□ avg (**Ω/□**)	Thickness avg (nm)	Conductivity(S/cm)
1	8178	236 ± 24	4.6 ± 0.5
2	10178	204 ± 17	4.8 ± 0.4
3	8538	219 ± 8	5.3 ± 0.2

The conductivity measurements were obtained from sheet resistance ($R_\square$ $or\ R\ square$) and film thickness (t). To measure $R_\square$, the Van der Pauw technique was used; probes were placed at each of the four corners of a square sample, with current flowing along one side (two probes sharing an edge) and the voltage being measured across the opposite edge. Then, the measurement is repeated after rotating the contacts 90º. The in-home set-up uses Labview to fit the resistance values from the slope of the I-V curved to the Van der Pauw equation

$$\left(e^{\left(-\frac{\pi R_A}{R_\square}\right)} + e^{\left(-\frac{\pi R_B}{R_\square}\right)} = 1\right) \quad (1)$$

to obtain $R_\square$. The second value, thickness, was obtained by profilometry (Dektak 150 stylus profilometer). At least three values on different locations of the film were used to report an average value. All reported values from Table S1 were results of multiple samples averaged and error propagated to conductivity.

Additional conductivity measurements were conducted on a variety of DDB-F_{72} concentrations. The UV-Vis spectra of varying concentrations of DDB-F_{72} is shown in Figure S1, where the bandgap peak goes down with increasing dopant concentration, and P1 and P2 increases with increasing concentration, indicating a more doped polymer. Moreover, conductivity measurements were conducted on these films showing an increase of conductivity with increasing dopant concentration.

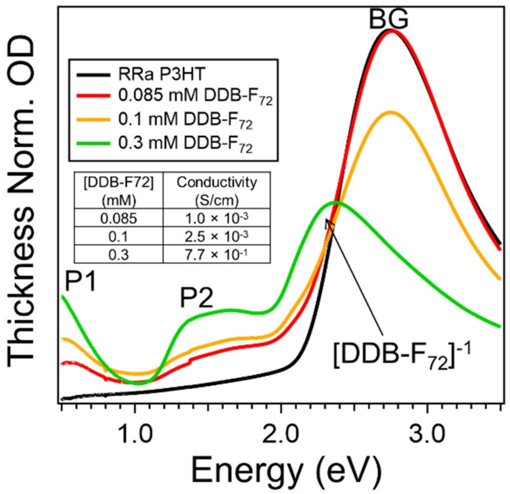

Figure S1. UV-Vis absorbance spectra of RRa P3HT doped with varying concentrations of DDB-F_{72}. Doping is characterized by the bleaching of the P3HT neutral peak (~2.8 eV) corresponding to the bandgap transition and the appearance of polaron transitions in the red and IR ranges

(designated P1 & P2) and the DDB-F_{72} anion absorption. The conductivity values of these films are shown to increase with increasing dopant concentration.

2. Neutron Reflectometry (NR) & Doping Efficiency Calculation

The active layer scattering length density (SLD) is the average of the SLD of all of the components. Since the SLD for the active layer and the components are known either by measurement or calculation, the SLD fraction can be calculated as shown in Table S2.

Table S2. NR calculation for DDB-F_{72} doped RRa P3HT

	Active Layer (AL)	RRa P3HT in AL	DDB-F_{72} in AL
SLD	1.36 (Measured)	0.45 (Measured)	2.0 (Calculated)
SLD Fraction	1	0.41	0.59
Monomer Ratio	N/A	7	1

P3HT has 25 atoms per monomer, DDB-F_{72} has 264 atoms per molecule. Using the SLD fraction, we can calculate the ratio of DDB-F_{72}:RRa P3HT monomer is 1:734 (1:7).

Dopant density in Table 1 is calculated based on the RRa P3HT monomer: DDB-F_{72} molecule ratio. We can calculate the number of RRa P3HT monomers in the pristine polymer film using RRa P3HT film density, film thickness, film area. The number of the RRa P3HT monomers is constant during the doping process, thus the number of DDB-F_{72} molecules (dopant density) can be calculated using the previous calculated dopant:monomer ratio.

Hole mobilities (μ_h) are directly measured from the AC Hall measurement. The DDB-F_{72} concentration used in the AC-Hall measurement is 1 mM. The dopant concentration used in NR is 0.85 mM since higher dopant concentration makes the film too rough to get a reasonable fitting. If we assume the mobile carrier mobility measured by AC Hall is the same as in NR, we can use μ_h and conductivities (σ) measured using the same sample sets as NR to calculate the hole densities (n_{Hall}) using the following equation;

$$doping\ efficiency = \frac{n_{Hall}}{dopant\ density} \quad (2)$$

The comparison NR data of RR and RRa P3HT are shown in Figure S1a and the SLD profiles in Figure S1b. The SLD of RR P3HT is slightly higher than that of RRa P3HT (0.62×10^{-6}Å^{-2} and 0.45×10^{-6}Å^{-2}, respectively). Additionally, as corroborated by profilometry, the thickness between the two films is different, with RR P3HT being thicker than RRa P3HT. When doped, this trend continues still, with RR P3HT (doped with a lower concentration at 0.3 mM DDB-F_{72}) being thicker and higher SLD. Additionally, both RR and RRa P3HT have a DDB-F_{72}-rich layer at the substrate-film interface. The RR P3HT data shown in Fig. S1a, b is previously published.[1]

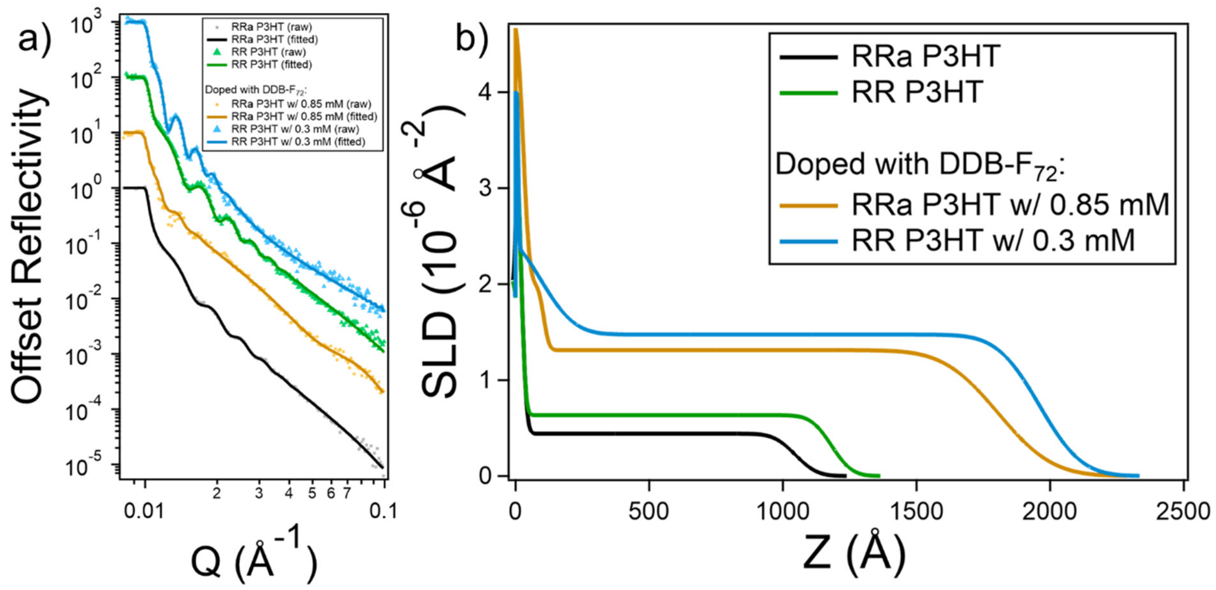

Figure S1. (a) Raw (markers) and fitted (solid line) neutron reflectometry (NR) profiles of undoped RRa P3HT (black trace), undoped RR P3HT (green trace), RRa P3HT doped with 0.85 mM DDB-F_{72} (yellow trace), and RR P3HT doped with 0.3 mM DDB-F_{72}(blue trace). (b) SLD profiles of undoped RRa P3HT (black trace), undoped RR P3HT (green trace), RRa P3HT doped with 0.85 mM DDB-F_{72} (yellow trace), and RR P3HT doped with 0.3 mM DDB-F_{72} (blue trace) Other than a slight buildup of DDB-F_{72} near the substrate surface (Z = 0 Å), DDB-F_{72} distributes uniformly in the bulk of both the RRa and RR P3HT films.

3. GISAXS for DDB-F_{72} doped P3HT

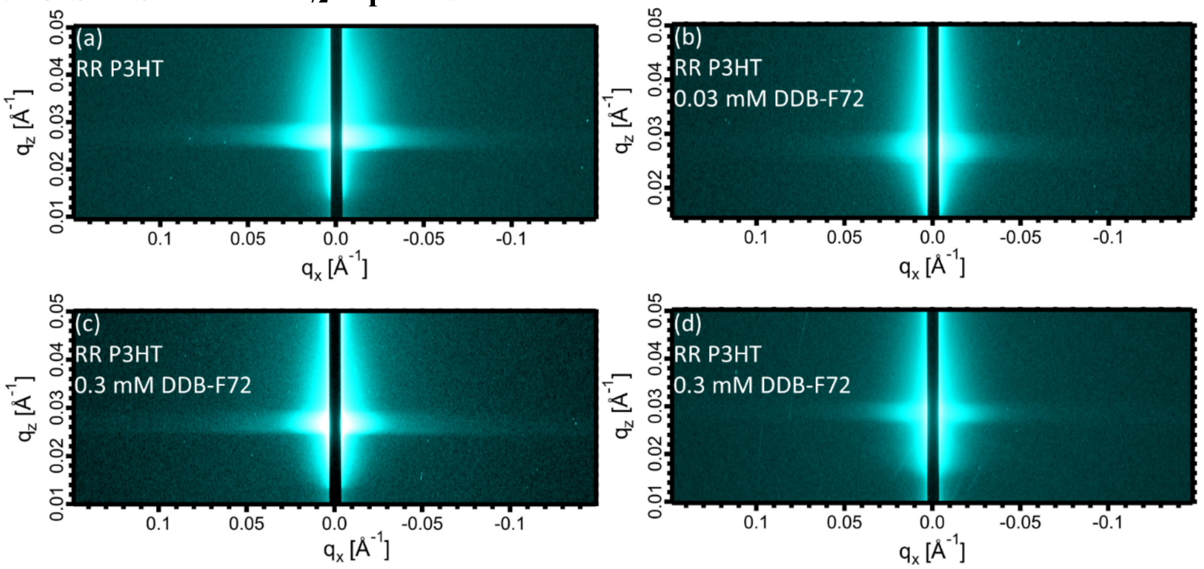

Figure S3. 2-D GISAXS for (a) RR P3HT, (b) RR P3HT doped with 0.03 mM DDB-F_{72}, (c) RR P3HT doped with 0.3 mM DDB-F_{72}, and (d) RR P3HT doped with 1 mM DDB-F_{72}.

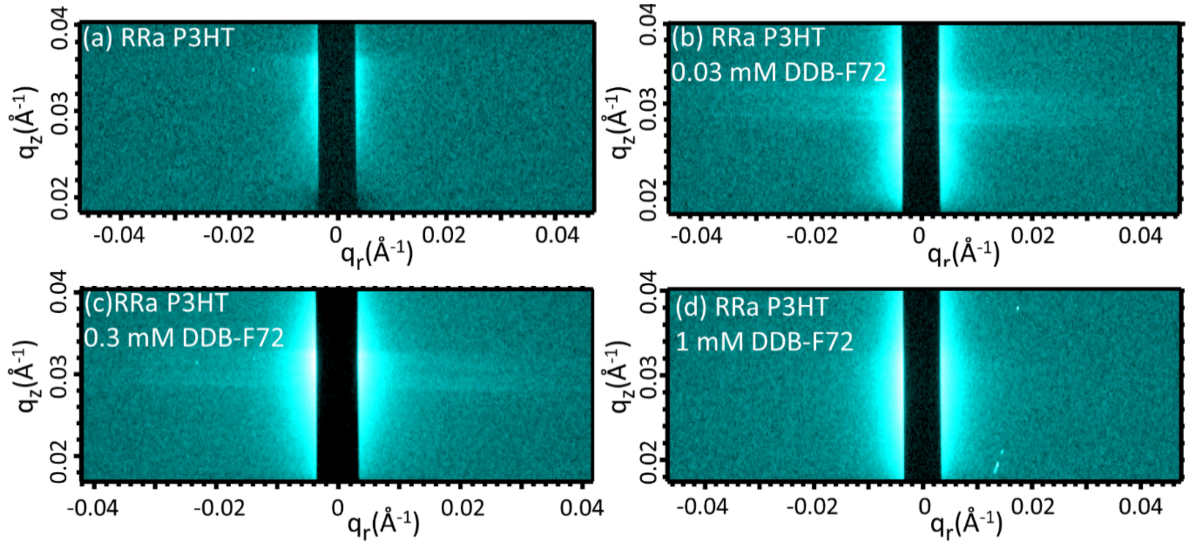

Figure S4. 2-D GISAXS for (a) RRa P3HT, (b) RRa P3HT doped with 0.03 mM DDB-F_{72}, (c) RRa P3HT doped with 0.3 mM DDB-F_{72}, and (d) RRa P3HT doped with 1 mM DDB-F_{72}.

4. GIWAXS for P3HT films doped with DDB-F_{72} at various dopant concentrations

2-D GIWAXS for DDB-F_{72} doped RR P3HT with more concentrations can be found in the supporting information in the previous work.[2] Below are the 2-D GIWAXS for DDB-F_{72} doped RRa P3HT and RR P3HT for this work.

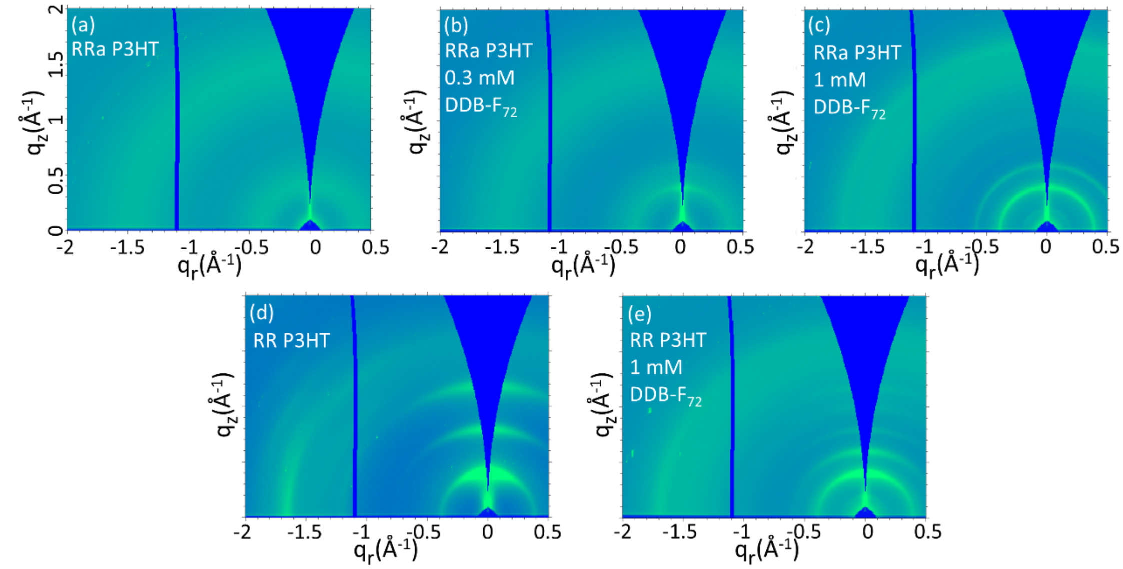

Figure S5. 2-D GIWAXS for (a) RRa P3HT, (b) RRa P3HT doped with 0.3 mM DDB-F_{72}, (c) RRa P3HT doped with 1 mM DDB-F_{72}, (d) RR P3HT and (e) RR P3HT doped with 1 mM DDB-F_{72}. For the amorphous RRa P3HT, crystalline structures start to form at 0.3 mM DDB-F_{72} doping level as shown by the appearance of lamellar overtones. Doping with DDB-F_{72} is shown to induce crystallinity in RRa P3HT polymer film, producing a similar crystalline structure in both RR and RRa P3HT at 1 mM dopant concentration.

5. Simulation Details for RR and RRa P3HT

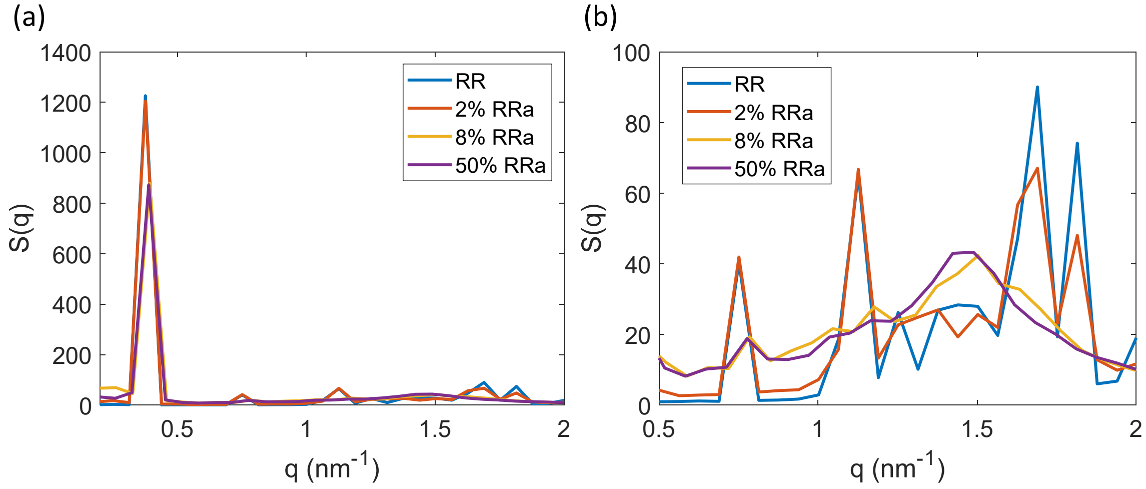

Figure S6: Calculated structure factor for RRa P3HT. The Percent RRa indicates the fraction of thiophene units that have the hexyl side chain attached to the other carbon, i.e. for 50% RRa, half of the thiophene units have their hexyl side chain attached to the C2 carbon rather than the C3 carbon (Scheme S1). (b) shows the broad peak at 1.5 q.

5.1 Additional Simulation Methods

MD simulations and calculations were carried out using the GROMACS package.[3-6] DFT calculations were carried out using Gaussian[7] with PBE0-D3/6-31G(d,p). The parameters for the forcefield and the charges are listed below. The parameters for the monomers whose thiophene units are flipped are not changed. Identical dihedral angle potential for the inter-thiophene bond is used for all monomers. All the simulations were carried out with periodic boundary conditions in all three axes in the isothermal-isobaric (NPT) ensemble using the Berendsen thermostat with $\tau_p = 5.0$ ps. The pressure is set at 250 bar, respectively, to match the experimental density of pure P3HT films. A timestep of 1 fs and a leap-frog integration algorithm were used. Electrostatics were treated using the Fast smooth Particle-Mesh Ewald (SPME) with cutoff at 1.4 nm, Fourier spacing of 0.12 nm, and PME order of 4. The van der Waals cutoff was set at 1.4 nm. For thermodynamic integration, a soft-function with $\sigma = 0.3$ and $\alpha = 0.5$ is used for lambda. An example mdp file, which sets all the MD parameters, is also included. Each system is annealed starting at 500 K at 2.5 ns, then at 400 K at 2.5 ns. Finally, at 300 K for 5 ns. The structure factors are calculated using the last ns of the trajectory. Structure factor is calculated using GROMACS's build-in function which uses an FFT algorithm.

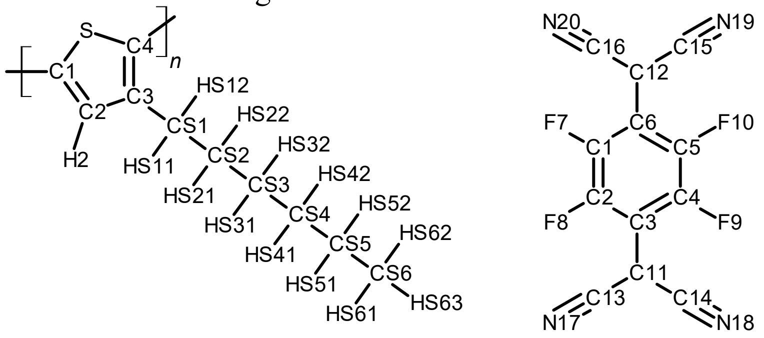

Scheme S1 Atom labels for the forcefield parameters

Table S4 Lennard-Jones parameters

	σ (nm)	ϵ (kJ mol^{-1})
P3HT		
S	0.360	0.355
C1, C2, C3, C4	0.355	0.355
H2	0.242	0.242
CS1, CS2, CS3,<br>CS4, CS5, CS6	0.350	0.350
HS11, HS12, HS21, HS22,<br>HS31, HS32, HS41, HS42,<br>HS51, HS52, HS61, HS62, HS63	0.250	0.250

Table S5 Bond Parameters

	b_0 (nm)	k_b (kJ mol^{-1} nm^{-2})
P3HT		
S-C1	0.1743	0.1734
S-C4	0.1725	0.1734
C1-C2	0.1367	0.1374
C1-C4	0.1458	0.1450
C2-C3	0.1430	0.1433
C2-H2	0.1084	0.1082
C3-C4	0.1374	0.1374
C3-CS1	0.1501	0.1509
CS1-HS11, CS1-HS12	0.1092	0.1098
CS1-CS2, CS2-CS3, CS3-CS4,<br>CS4-CS5, CS5-CS6	0.1529	0.1542
CS2-HS21, CS2-HS22,<br>CS3-HS31, CS3-HS32,<br>CS4-HS41, CS4-HS42,<br>CS5-HS51, CS5-HS52,	0.1090	0.1095

CS6-HS61, CS6-HS62, CS6-HS63		

Table S6 Angle Parameters

	θ_0 (deg)	k_θ (kJ mol^{-1} rad^{-2})
P3HT		
C1-S-C4	91.6	92.77
S-C1-C2	110.3	110.3
S-C4-C3	111.2	110.3
S-C1-C4	120.4	119.6
C2-C1-C4	128.4	130.1
C3-C2-H2	123.3	123.7
C1-C2-C3	114.0	113.3
C2-C3-C4	111.8	113.3
C1-C2-H2	122.6	123.0
C2-C3-CS1	122.8	123.4
C4-C3-CS1	125.4	124.5
C3-CS1-HS11, C3-CS1-HS12	111.3	109.2
C3-CS1-CS2	115.6	115.4
CS1-CS2-CS3, CS2-CS3-CS4, CS3-CS4-CS5, CS4-CS5-CS6	112.7	112.7
HS11-CS1-HS12	108.6	107.8
HS21-CS2-HS22, HS31-CS3-HS32, HS41-CS4-HS42, HS51-CS5-HS52, HS61-CS6-HS62, HS61-CS6-HS63, HS62-CS6-HS63	107.8	107.8
HS11-CS1-CS2, HS12-CS1-CS2, CS1-CS2-HS21, CS1-CS2-HS22, HS21-CS2-CS3, HS22-CS2-CS3, CS2-CS3-HS31, CS2-CS3-HS32, HS31-CS3-CS4, HS32-CS3-CS4, CS3-CS4-HS41, CS3-CS4-HS42, HS41-CS4-CS5, HS42-CS4-CS5, CS4-CS5-HS51, CS4-CS5-HS52,	110.7	110.7

HS51-CS5-CS6, HS52-CS5-CS6, CS5-CS6-HS61, CS5-CS6-HS62, CS5-CS6-HS63	

Table S7 Dihedral Parameters

(kJ mol^{-1})	C0	C1	C2	C3	C4
C2-C1-S-C4, S-C1-C2-H2, S-C1-C2-C3, C1-S-C4-C3, C1-S-C4-C1, C1-C2-C3-CS1, C1-C2-C3-C4, H2-C2-C3-CS1, H2-C2-C3-C4, C2-C3-C4-S, C2-C3-C4-C1, CS1-C3-C4-S, CS1-C3-C4-C1, C4-C1-S-C4, C4-C1-C2-H2, C4-C1-C2-C3	30.33	0	–30.33	0	0
C2-C3-CS1-HS11, C2-C3-CS1-HS12, C2-C3-CS1-CS2, C4-C3-CS1-HS11, C4-C3-CS1-HS12, C4-C3-CS1-CS2	0	0	0	0	0
C3-CS1-CS2-HS21, C3-CS1-CS2-HS22	0.9665	2.900	0	–3.866	0
C3-CS1-CS2-CS3	2.929	–1.464	0.2092	–1.674	0
HS11-CS1-CS2-HS21, HS11-CS1-CS2-HS22, HS11-CS1-CS2-CS3, HS12-CS1-CS2-HS21, HS12-CS1-CS2-HS22, HS12-CS1-CS2-CS3, CS1-CS2-CS3-HS31, CS1-CS2-CS3-HS32, CS1-CS2-CS3-CS4, HS21-CS2-CS3-HS31, HS21-CS2-CS3-HS32, HS21-CS2-CS3-CS4, HS22-CS2-CS3-HS31, HS22-CS2-CS3-HS32, HS22-CS2-CS3-CS4, CS2-CS3-CS4-HS41, CS2-CS3-CS4-HS42, CS2-CS3-CS4-CS5, HS31-CS3-CS4-HS41, HS31-CS3-CS4-HS42, HS31-CS3-CS4-CS5, HS32-CS3-CS4-HS41, HS32-CS3-CS4-HS42, HS32-CS3-CS4-CS5, CS3-CS4-CS5-HS51, CS3-CS4-CS5-HS52, CS3-CS4-CS5-CS6, HS41-CS4-CS5-HS51, HS41-CS4-CS5-HS52, HS41-CS4-CS5-CS6, HS42-CS4-CS5-HS51, HS42-CS4-CS5-HS52, HS42-CS4-CS5-CS6, CS4-CS5-CS6-HS61, CS4-CS5-CS6-HS62, CS4-CS5-CS6-HS62, HS51-CS5-CS6-HS61, HS51-CS5-CS6-HS62, HS51-CS5-CS6-HS63, HS52-CS5-CS6-HS61,	0.6276	1.883	0	–2.510	0

HS52-CS5-CS6-HS62, HS52-CS5-CS6-HS63					
S-C4-C1-S, C3-C4-C1-C2	0.4377	–0.3997	–3.027	1.712	–0.8584
S-C4-C1-C2, C3-C4-C1-S	0.4377	0.3997	–3.027	–1.712	–0.8584
C4-C3-S-C1 (imprope)	180	4.602	–	–	–

Table S8 Partial Charges

	q
P3HT	FF1
S	–0.011
C1	0.118
C2	–0.260
H2	0.155
C3	0.078
C4	–0.118
CS1	–0.114
HS11, HS12	0.053
CS2	0.074
HS21, HS22	–0.005
CS3	–0.036
HS31, HS32	0.002
CS4	–0.014
HS41, HS42	0.002
CS5	0.172
HS51, HS52	–0.031
CS6	–0.215
HS61, HS62, HS63	0.043

Below is the mdp file used for the calculation.

```
title              =  cpeptid position restraining
cpp                =  /usr/bin/cpp
constraints        =  none
integrator         =  md
dt                 =  0.001    ; ps !
nsteps             =  10000000      ; total 1.0 ps.
nstcomm            =  100
nstxout            =  10000
nstvout            =  10000
nstfout            =  0
nstlog             =  100
nstenergy          =  100
nstlist            =  100
ns_type            =  grid
rlist              =  1.0
coulombtype        = PME
vdwtype            = cut-off
rcoulomb           =  1.0
rvdw               =  1.0
fourierspacing     = 0.12
fourier_nx         = 0
fourier_ny         = 0
fourier_nz         = 0
pme_order          = 4
ewald_rtol         = 1e-5
optimize_fft       = yes
pbc                = xyz
periodic-molecules  = yes
; Berendsen temperature coupling is on
Tcoupl = v-rescale
tau_t = 0.1
tc-grps  =system
ref_t =   300
; Pressure coupling is  on
Pcoupl             = berendsen
pcoupltype         = isotropic
; Pcoupl           =  no
tau_p              =  5.0
compressibility    =  4.5e-5
ref_p              =  250.0
; Generate velocites is on at 300 K.
gen_vel            =  yes
gen_temp           =  500.0
; Anneal
```

```
annealing          =  single
annealing-npoints   =  3
annealing-time      =  0 2500 5000
annealing-temp      =  500 400 300
```

References:

1. Aubry, T. J., Winchell, K. J., Salamat, C. Z., Basile, V. M., Lindemuth, J. R., Stauber, J. M., Axtell, J. C., Kubena, R. M., Phan, M. D., Bird, M. J., Spokoyny, A. M., Tolbert, S. H., Schwartz, B. J. "Tunable Dopants with Intrinsic Counterion Separation Reveal the Effects of Electron Affinity on Dopant Intercalation and Free Carrier Production in Sequentially Doped Conjugated Polymer Films," *Adv. Funct. Mater.* **2020**, *30*, 2001800.

2. Aubry, T. J., Axtell, J. C., Basile, V. M., Winchell, K. J., Lindemuth, J. R., Porter, T. M., Liu, J. Y., Alexandrova, A. N., Kubiak, C. P., Tolbert, Spokoyny, A. M., Schwartz, B. J. "Dodecaborane-Based Dopants Designed to Shield Anion Electrostatics Lead to Increased Carrier Mobility in a Doped Conjugated Polymer," *Advanced Materials* **2019**, *31*, 1805647.

3. Lindahl, E.; Hess, B.; van der Spoel, D. GROMACS 3.0: A Package for Molecular Simulation and Trajectory Analysis Received: *Journal of Molecular Modeling* **2001**, *7*, 306–317..

4. van der Spoel, D.; Lindahl, E.; Hess, B.; Groenhof, G.; Mark, A. E.; Berendsen, H. J. C. GROMACS: Fast, Flexible, and Free. *Journal of Computational Chemistry* **2005**, *26* (16), 1701–1718.

5. Hess, B.; Kutzner, C.; van der Spoel, D.; Lindahl, E. GROMACS 4: Algorithms for Highly Efficient, Load-Balanced, and Scalable Molecular Simulation. *Journal of Chemical Theory and Computation* **2008**, *4* (3), 435–447.

6. Pronk, S.; Pall, S.; Schulz, R.; Larsson, P.; Bjelkmar, P.; Apostolov, R.; Shirts, M. R.; Smith, J. C.; Kasson, P. M.; van der Spoel, D.; Hess, B.; Lindahl, E. GROMACS 4.5: A High-Throughput and Highly Parallel Open Source Molecular Simulation Toolkit. *Bioinformatics* **2013**, *29* (7), 845–854

7. Gaussian 09, Revision A.02, M. J. Frisch, G. W. Trucks, H. B. Schlegel, G. E. Scuseria, M. A. Robb, J. R. Cheeseman, G. Scalmani, V. Barone, G. A. Petersson, H. Nakatsuji, et al., Gaussian, Inc., Wallingford CT, 2016.
